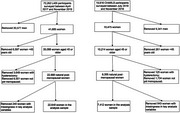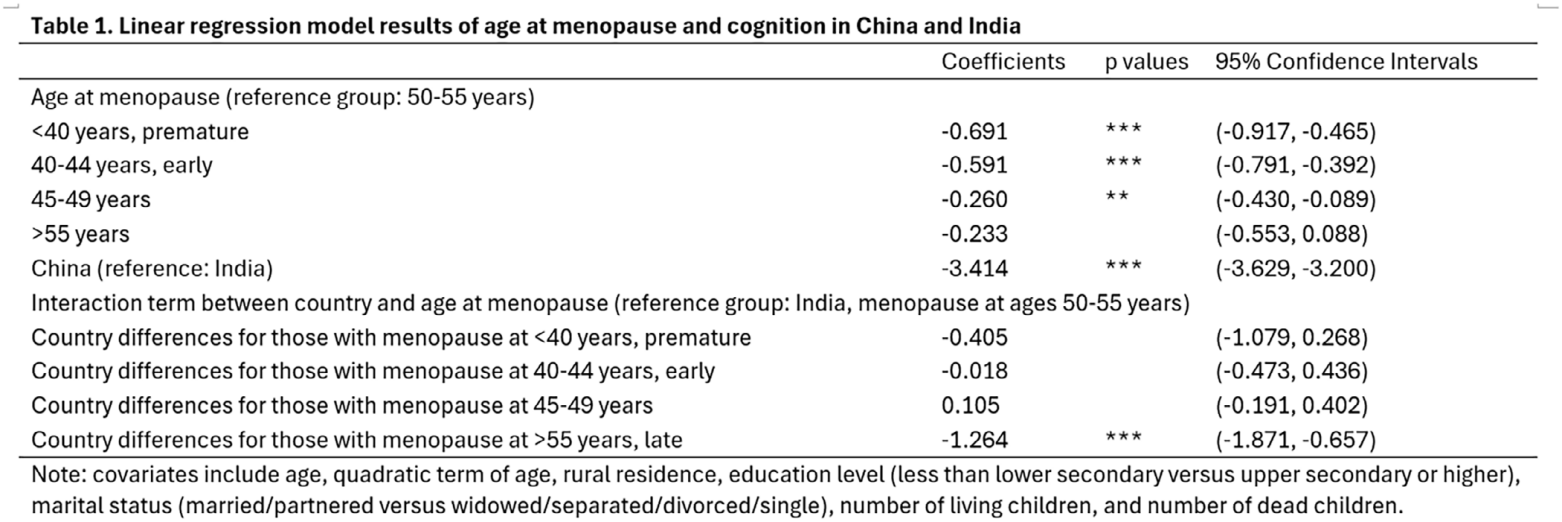# Age at menopause and cognition in later life – a cross‐nation comparison study between China and India

**DOI:** 10.1002/alz70860_103939

**Published:** 2025-12-23

**Authors:** Muqi Guo

**Affiliations:** ^1^ University of Michigan School of Public Health, Ann Arbor, MI, USA

## Abstract

**Background:**

Women have a higher prevalence of dementia than men. While midlife risk factors have been studied, they do not fully explain sex differences in dementia. Age at menopause, marked by a sharp drop in estrogen with neuroprotective effects, may influence later‐life cognitive function. This study investigates this relationship, comparing women in China and India, regions with limited evidence and no prior cross‐national analyses.

**Method:**

We analyzed nationally representative samples of postmenopausal women aged 45+ from the China Health and Retirement Longitudinal Study (CHARLS, 2018) and the Longitudinal Aging Study in India (LASI, 2017–2019). The sample included 7,412 Chinese and 22,648 Indian women. Menopause age was categorized into <40, 40–44, 45–49, 50–55, and >55 years. Cognitive function was assessed via composite scores from neuropsychological tests. Linear regression models examined the association between menopause age and cognition, adjusting for demographics (country, age, education, rural residence, marital status, and number of children). Interaction terms tested whether associations differed by country.

**Result:**

Indian women were younger (mean age 61.4 vs. 63.0) and had higher education levels (16% vs. 8.5%). Premature (<40) and early menopause (40–44) were more common in India (12.7% and 20.8%) than China (3.2% and 8.4%). Conversely, Chinese women experienced menopause more often at 50–55 years (49.5% vs. 20.8%). Cognitive scores were lower in Chinese women. Across both countries, younger menopause ages (<50) were linked to poorer cognition. In India, no significant cognitive differences were observed between menopause at 50‐55 years and after 55 years. However, in China, late menopause (>55) was associated with worse cognitive function.

**Conclusion:**

Earlier menopause is associated with worse cognitive function. Indian women face a greater burden due to higher prevalence of premature and early menopause, while Chinese women are additionally affected by lower cognitive function associated with late menopause.